# Diagnostic Application of Targeted Next-Generation Sequencing of 80 Genes Associated with Disorders of Sexual Development

**DOI:** 10.1038/srep44536

**Published:** 2017-03-15

**Authors:** Yanjie Fan, Xia Zhang, Lili Wang, Ruifang Wang, Zhuo Huang, Yu Sun, Ruen Yao, Xiaodong Huang, Jun Ye, Lianshu Han, Wenjuan Qiu, Huiwen Zhang, Lili Liang, Xuefan Gu, Yongguo Yu

**Affiliations:** 1 Department of Pediatric Endocrinology/Genetics, Xinhua Hospital, Shanghai Jiao Tong University School of Medicine, Shanghai Institute for Pediatric Research, Shanghai, 200092, China; 2 Department of Endocrinology, Shanghai Children’s Medical Center, Shanghai Jiao Tong University School of Medicine, Shanghai, 200127, China

## Abstract

Disorders of sexual development (DSD) are estimated to occur in 1 of 4500 births. Since the genetic etiology of DSD is highly heterogeneous, obtaining a definitive molecular diagnosis by single gene test is challenging. Utilizing a high-throughput sequencing upfront is proposed as an efficient approach to aid in the diagnosis. This study aimed to examine the diagnostic yield of next-generation sequencing in DSD. 32 DSD patients that previously received clinical examinations and single gene tests were selected, with or without a diagnosis. Prior single gene tests were masked, and then samples went through targeted next-generation sequencing of 80 genes from which the diagnostic yield was assessed. A likely diagnosis, with pathogenic or likely pathogenic variants identified, was obtained from nine of the 32 patients (i.e., 28.1%, versus 10% by single gene tests). In another five patients (15.6%), variants of uncertain significance were found. Among 18 variants identified (i.e., 17 single nucleotide variants and one small deletion), eight had not been previously reported. This study supports the notion that next-generation sequencing can be an efficient tool in the clinical diagnosis and variant discovery in DSD.

Sexual development is an orchestrated pathway that directs undifferentiated zygotes towards male or female destiny^1^. Disruption of the tightly regulated process of sex determination and differentiation can result in disorders of sexual development (DSD), defined as a state when the “development of chromosomal, gonadal or anatomical sex is atypical”^2^. The genetic causes of DSD are highly heterogeneous, complicated by the associated genetic alterations that include copy number variations (CNVs), single nucleotide variations (SNVs) and small insertions and deletions (InDels)^3^.

Etiological diagnosis of DSD usually requires a wide spectrum of endocrinological tests, radiological imaging and genetic tests^2^. Traditionally, clinicians conduct physical examinations, endocrine tests and radiological imaging in the first instance, and then request single genetic tests to confirm the diagnosis. Thus far, no study has yet been published regarding the diagnostic yield of Sanger sequencing following this traditional routine in a large DSD cohort with mixed conditions. Sporadic studies on specific DSD condition hint that the yield might be low. For example, a study in Korean patients with Kallmann syndrome, which is a relatively common DSD seen in the endocrinology clinic, showed that molecular defects were found in 16% of the cohorts after a thorough endocrine/radiological workup and Sanger sequencing of 10 genes^4^. The actual yield in the practice is probably lower, considering the single gene tests requested for a specific condition are usually less than 10. This is also a lengthy process. It could be very stressful when medical treatments or surgical decisions are pending. To address these challenges, one proposal is to initially sequence all the current known genes of DSD^5^, and then conduct more specific endocrine and clinical tests guided by the obtained genetic results.

Recent advances in next-generation sequencing have greatly accelerated the discovery of genetic variants. The Vilain lab conducted two studies, one of which, was a targeted panel sequencing of 35 DSD genes that revealed genetic defects in two of the seven patients not previously diagnosed, and confirmed the diagnosis in another seven patients with known genetic causes^6^. In another study, whole exome sequencing followed by analysis of selected DSD genes reached a diagnostic yield of 22.5% in 40 patients presenting with 46XY DSD^7^. A more recent study by the Liang lab showed a relatively higher yield by a targeted panel sequencing approach, in which a genetic diagnosis in eight out of 21 (38.1%) DSD patients was obtained, with patients harboring copy number variations included^8^. These studies demonstrated the diagnostic potential of a high-throughput approach like next-generation sequencing (NGS).

In this study, we designed a targeted panel to sequence 80 genes, including known genes associated with human DSD and some recently identified genes that influence the pathways of sex determination or differentiation. Thirty-two Chinese patients with DSD were recruited, each of whom had been subjected to the traditional approach of endocrine analysis and radiological imaging followed by single genetic tests. Their prior genetic testing results were blinded in the sample selection, thus inclusion in this study was not predicated on whether a diagnosis was previously established. The diagnostic yield was compared between the single genetic test and high-throughput sequencing to evaluate the potential of NGS utilization in a clinical setting.

This current study aimed to investigate the diagnostic potential of high-throughput sequencing in a mixed cohort of DSD, to provide the mutation spectrum of DSD patients in the Chinese population, and to potentially discover novel genetic variants in DSD.

## Materials and Methods

### Subject selection

Subjects were selected from patients visiting the pediatric endocrinology clinic at Xinhua hospital or the Children’s medical center, Shanghai, China during August to December 2014. Most patients were from Eastern China. The recruitment was based on the clinical criteria of DSD diagnosis^9^. The inclusion required consent from patients and family, complete clinical records and access to prior results of biochemical, radiological and single genetic tests (if requested). Karyotype analysis was done in all patients as routine. Those patients with chromosomal abnormality known to cause the DSD phenotype, for example 45,XO Turner’s syndrome, Klinefelter’s syndrome, dup Xp21.3 (with the NR0B1 gene duplication known to cause 46,XY complete gonadal dysgenesis) and others were excluded. Subjects with a clear indication of classic congenital adrenal hyperplasia were also excluded. Subjects may have gone through biochemical assays, ultrasound and other clinical workup. A total of seven pediatric endocrinologists were involved in this study, and most of the 32 patients were seen by two clinicians. Genetic tests by Sanger sequencing of single genes were requested following the clinician’s professional judgment (at least one main phenotype matches the genetic condition) on the most likely genetic causes. In subject selection and subsequent analysis, the results of all prior genetic tests were not revealed to the investigator. All patients selected were derived from unrelated families. The study was reviewed and approved by the ethical committee at Xinhua hospital, all methods were performed in accordance with the relevant guidelines and regulations, and informed consent was obtained from the patients and parents (i.e., for patients under 18 years old).

### Selection of candidate genes

The list of 80 DSD genes was generated by searching online database and the available literature including OMIM (http://www.omim.org), HGMD (http://www.hgmd.cf.ac.uk/) and Pubmed (http://www.ncbi.nlm.nih.gov/pubmed). The keywords that were used in this online search included: “gonadal”, “sex development”, “hypogonadism”, “testicular”, “ovarian”, “hormone” and their combinations with “disorder” or “disease”. The list was then curated to include those genes that were reported in at least one human DSD case, or that had multiple lines of evidence suggesting its role in sexual development based on the model organism (please see Table 1).

### Sample preparation, sequencing and analysis

Approximately 2 ml of peripheral venous blood was drawn from patients to standard K3EDTA-tubes, and genomic DNA was extracted with the GentraPuregene Kit (Qiagen, Germany) or the Lab-Aid 820 kit (ZSandx, China). Coding and flanking regions of the 80 DSD genes were amplified using a customized Ion Ampliseq panel (Life Technologies).

The preparation of each library was completed according to the manufacturer’s instructions, including automated template preparation by the Ion One-touch System. All sample preparations were then sequenced on the PGM with 316 or 318 chip (Ion PGM Sequencing 200 kit v2, Life technologies). Approximately 0.4G of raw data was obtained for each sample. Data analysis was performed on the Torrent server. Base calling, read filtering, alignment to the human genome (hg19), and variant calling were done with Ion Torrent Suites (Life technologies).

Picard (http://picard.sourceforge.net/) and the Genome Analysis Tool Kit (GATK, http://www.broadinstitute.org/gatk/) were used for further QC analysis, coverage analysis and variant filtering, and snpeff/snpsift (http://snpeff.sourceforge.net/) were used to annotate the variants. For basic filtering, variants with a coverage depth less than five-fold or a Phred-like score below 30 were excluded. Subsequently, variants with a frequency >1% in the Exome Aggregation Consortium (ExAC, http://exac.broadinstitute.org/), 1000 Genome Project (http://www.1000genomes.org/) or Exome Sequencing Project (ESP, http://evs.gs.washington.edu/EVS/), or >20% in our in-house database were excluded (based on 72 whole exome datasets and 130 datasets from Agilent Clearseq inherited disease panel). It should be noted that a large portion of our in-house datasets were from patients with endocrine disorders, and thus a high threshold was applied to avoid over-filtration due to the enrichment of DSD samples. Quality metrics were generated and evaluated (with the description found in the Results section 3.2). All variants of interest were visually inspected in the Integrative Genomics Viewer (https://www.broadinstitute.org/igv) to evaluate the mapping and variant calling quality. The candidate variants were then classified into five main categories using the American College of Medical Genetics and Genomics guidelines - pathogenic, likely pathogenic, variants of uncertain clinical significance (VUS), likely benign, and benign^10^. Sanger sequencing was performed to validate those variants categorized as pathogenic, likely pathogenic or VUS. Segregation was analyzed when parental samples were available.

## Results

### Clinical profiles and the diagnostic yield of prior single-gene tests in the traditional approach

We recruited 32 patients from the pediatric endocrinology clinic, including 27 patients with a 46,XY karyotype and five patients with a 46,XX karyotype. As listed in Table 2, the patients presented with a variety of DSD phenotypes. Among the 27 patients with 46,XY DSD, 20 patients presented primarily with micropenis and small testes, four patients with hypospadias, two patients with cryptorchidism, one patient with ambiguous genitalia, and one patient with 46,XY complete gonadal dysgenesis. Among the five patients with a 46,XX karyotype, the majority (4/5) of the patients presented with ambiguous genitalia. Anosmia was reported in three patients.

Sanger sequencing of single genes was ordered depending on the clinician’s judgment on the most likely genetic cause, following endocrine and radiological workup. The results of these prior genetic tests reflected the molecular diagnostic rate following traditional routines in our clinical practice (listed in Table 2 column “prior tests”). A total of 41 single gene tests by Sanger sequencing were requested for 30 patients, and pathogenic or likely pathogenic variants were identified in three patients (i.e., P01, P27, P32), obtaining a diagnostic rate of 10% (3/30). Both *NR0B1* and *KAL1* were the most frequently requested genetic tests, and all three patients with a likely diagnosis by single gene tests indeed harbored mutations in those genes. In this current study of NGS, information obtained from prior genetic tests was masked during subject selection and subsequent data analysis.

### Quality control, diagnostic yield and genetic variants identified by NGS

Key quality metrics were assessed to validate the performance of targeted panel sequencing. In this analysis, 98.6% of the reads were aligned to the reference genome, with an average sequencing depth of 185X on targeted regions. In addition, 94.6% of the targeted regions were covered by ten or more reads (please see Supplementary Table 1). All variants of interest were subsequently validated by Sanger sequencing.

Variants suspected to relate to the patient’s condition are listed in Table 3. A likely molecular diagnosis - with pathogenic or likely pathogenic variants identified - was obtained from 9 of the 32 patients (28%). These include three patients (i.e., P09, P22, P28) with 5-alpha-reductase deficiency due to biallelic mutations of the *SRD5A2* gene, and four patients with idiopathic hypogonadotropic hypogonadism due to mutations of *KAL1* (i.e., P27, P32), *PROKR2* (P03) or *GNRHR* (P13). In addition, one patient with hypogonadotropic hypogonadism and adrenal insufficiency (later treated by hydrocortisone) was found to harbor a hemizygous mutation of the *NR0B1* gene (P01), and one patient with androgen insensitivity syndrome was found to harbor a hemizygous mutation of the *AR* gene (P21). Therefore, the diagnostic yield of NGS (28%) in our study was almost three-fold that of the prior single gene tests (10%) as requested by clinicians in practice.

## Discussion

### Increased diagnostic yield of sequencing by the DSD panel as compared with single genes

Panel sequencing confirmed all three cases with a previous genetic diagnosis (3/3, 100%) and identified the genetic causes in six of the 29 patients that received negative results by prior analyses (6/29, 20.7%), reaching an almost three-fold increase in yield over the traditional single gene approach in our center’s practice (28.1% versus 10%). The detection rate of single gene test is largely influenced by the selection of target gene, which is limited by the clinicians’ knowledge and experience with each genetic condition. For disorders like DSD, the phenotypes do not differentiate well among different conditions, thus the selection of target gene could be subjective. Even though, the detection rate reflects the possible outcome of traditional approach in one center’s clinical practice. For this given mixed group of patients, NGS excels the single gene test in DSD diagnosis.

The diagnostic rate of NGS in our study is similar to the two studies by the Vilain lab (28% and 22.5%)^6^,^7^. Compared with the targeted panel by the Vilain lab, we included some genes known to be essential regulators of sex development but only few variants have been reported in human samples, like *WDR11, BMP4* and *SEMA3A*, and eventually identified novel variants of these genes in patients who remain undiagnosed (Table 3b). In a more recent study by the Liang lab in the Chinese population, the targeted panel included 219 genes. Excluding two patients with large copy number variations, likely genetic causes were identified in 6 out of 21 patients (~28.6%)^8^, which is close to our study.

Obtaining a diagnosis largely depends on variants identification in the well-established DSD genes - those genes with multiple lines of evidence to support their roles in pathogenesis. These well-established genes were basically covered by all four studies, including Vilain’s (two studies)^6^,^7^, Liang’s^8^ and our study. These four studies shared a core set of established genes and eventually reached a similar diagnostic rate (i.e., Vilain lab:28% & 22.5%, Liang lab: 28.6%, our group:28.1%). This also suggests 28% might be a possible limit of diagnostic rate based on currently established genes in human DSD, and further understanding of DSD etiology may expand the pool of candidate genes and eventually increase the diagnostic yield. Compared with the other three studies, our study provides the direct comparison of diagnostic rate between single gene tests following traditional approach (endocrine and radiological workup) and next-generation sequencing. The advantage of next-generation sequencing shown in this study supports a shift of paradigm in the clinical practice, based on a single center’s experience.

In a further analysis, we attempted to identify the overlapped target genes among those previously published studies^6^,^7^,^8^ and this current report. By deriving data that was obtained from these three studies (Vilain’s^6^, Liang’s^8^ and our study), we employed the website http://bioinformatics.psb.ugent.be/cgi-bin/liste/Venn to construct a Venn diagram (Please refer to Supplementary Figure S1) of these overlapped target genes and found 22 genes that were common between all three studies. In addition, we found 22 plus 34 overlapped genes when comparing our study and that study previously reported by Liang’s group^8^. Further, all of the detected mutations in our study (Table 3) were located in the 22 plus 34 overlapped gene group (please see Supplementary Figure S1). Most of these overlapped genes are generally agreed disease-causing genes in DSD. The observation that detected variants located in this group suggested that it would be suitable to select only the identified 22 plus 34 gene sets as an initial “quick and small” screening panel, for patients whose main phenotypes are micropenis, small testes, cryptorchidism or ambiguous genitalia. This panel could be continuously optimized with more NGS data collected and a broader mutation spectrum revealed. We recommend this approach over the previously applied approach of attempting to analyze a large number of target genes such as the 80 gene set in our study. Clearly, this would afford a significant technical work effort and financial cost saving usually invested in the diagnosis of DSD. It should be mentioned that copy number variations were not examined in this study. A more comprehensive genetic panel including copy number variations may reveal another 13–22% of disease etiology, based on previous reports of chromosome microarray analysis^11^,^12^.

### Frequent genetic causes in Chinese DSD patients

The mutational spectrum of DSD genes has not been well characterized in a Chinese population. For those patients presenting under-virilization, the genetic tests most often prescribed in our clinic are *NR0B1* and *KAL1*. This reflects the “hot” candidate genes in clinicians’ opinion, which could be biased by their previous experience with those genetic conditions. Our results revealed *SRD5A2* variants were the most frequent in the study group - with five variants identified in 3 patients. This is followed by *KAL1* with 2 variants identified. *NR0B1, GNRHR, AR*, and *PROKR2* variants were identified in one patient each. 5-alpha-reductase deficiency appears to be the most common defect, though not always the top possible etiology our clinicians initially consider before Dihydrotestosterone/Testosterone measurements. Future studies regarding the prevalence of each DSD condition would offer the clinicians a prioritized list of endocrine tests in the practice.

### Possible genotype-phenotype correlation of *SRD5A2* mutations

An interesting observation was that the severity of undervirilization appears to correlate with the genotype of *SRD5A2* in our **three** 46, XY DSD patients. Frameshift or nonsense variants usually exert more detrimental effects on the enzymatic function than missense variants. P09 harbors two missense variants (p.Arg227Gln and p.Gly203Ser) and presents a relatively mild phenotype - micropenis and small testes. The variant p.Arg227Gln is reported to retain ~3.2% enzyme activity and associate with micropenis in compound heterozygous state^13^. The phenotype of P09 is consistent with literature. However, P28, who harbors this missense variant (p.Arg227Gln) and a frameshift variant (p.Phe219fs), presents a moderate degree of undervirilization - hypospadias in addition to micropenis. P22, who harbors a homozygous nonsense variant (p.Gln6*), exhibits the most severe phenotype of these three patients - presenting a female appearance and having ambiguous genitalia with testis on the right and a vagina. This might hint an increasing degree of undervirilization correlates with the more deleterious variants of *SRD5A2*, though enzymatic function assay of steroid 5-alpha-reductase and larger sample numbers are needed to validate this correlation. Nonetheless, this example clearly shows the broad range of phenotypic variations exerted by mutations of the same DSD gene.

### Novel variants identified in DSD genes

Genes involved in the fine-tuned process of sex determination and differentiation have been studied in many model organisms, while not all the genes have been reported with human mutations. Inclusion of these genes in the panel sequencing may reveal variants in those lesser-known DSD genes.

*DMRT1* is a gene exclusively expressed in male gonads^14^. This gene was originally identified in chromosome 9p, the critical region of which was lost in 9p monosomy patients with defective testis development and 46,XY complete gonadal dysgenesis^15^. So far, very few variants have been reported in this gene. Murphy *et al*. reported the first point mutation of *DMRT1* (p.R111G) in a 46,XY complete gonadal dysgenesis patient, and showed the heterozygous mutant affects DNA binding affinity and results in a severe phenotype^16^. Similar to the patient with p.R111G mutation, our patient P19, who has a 46, XY karyotype with normal SRY, harbors a novel missense variant c.251 A > G (p.Try84Cys) and exhibits complete gonadal dysgenesis. This heterozygous variant was transmitted from the unaffected mother. As DMRT1 is only expressed in male-specific gonads, it is possible that the defects of DMRT1 do not affect ovarian development, thus female carriers are normal and fertile.

*WDR11* is considered to associate with hypogonadotropic hypogonadism with or without anosmia (OMIM#614858). Five missense variants were reported so far, all from one study^17^. Our patient P06 harbors a novel missense variant c.817C > G (p.Leu273Val, predicted to be deleterious) and presents the phenotype of idiopathic hypogonadotropic hypogonadism with small testes and normosmia. This is consistent with the phenotypes in literature^17^. However, unlike the missense variants previously reported, this variant is not located in the region of WD-domain, which is important for protein-protein interaction. Further evidence is needed to verify if the variant indeed affects the production of WDR11 or protein interaction in pubertal development.

*BMP4* is a member of the bone morphogenetic protein (BMP) family that plays a vital role in embryonic development. There were 4 missense variants of BMP4 reported in 46,XY DSD patients with hypospadias^18^, and BMP4 is thought to regulate the signaling cascades involved in urethral development. A missense variant c.806G > A (p.Arg269Gln) was detected in our patient P07, with a 46, XX karyotype. P07 has a male appearance with hypospadias and inguinal testes. The *BMP4* association with 46, XX testicular DSD has not been reported before. It is possible that the *BMP4* variant affects the urethral formation in our patient, and another unidentified genetic variant causes the sex reversal phenotype. Based on current evidence, the clinical significance of the *BMP4* variant identified in our patient cannot be determined.

Variations of *SEMA3A* and *AKR1C4* are both implicated in DSD, but definite roles are not established. *SEMA3A* variants were reported in patients of hypogonadotropic hypogonadism (OMIM#614897). Based on the normal phenotype of heterozygous mutant mice, mono-allelic mutation of *SEMA3A* may not be sufficient to cause sex differentiation problems without synergistic effect of another mutant allele of DSD genes^19^. The heterozygous *AKR1C4* variant has been previously reported in a Swiss family of 46,XY complete gonadal dysgenesis^20^. We found a missense variant of *SEMA3A* (c.487G > C, p.Glu163Gln) and a missense variant of *AKR1C4* (c.773G > A, p.Arg258His) both present in P23, who showed ambiguous genitalia including labioscrotal hypertrophy, micropenis and inguinal testes. This patient had a 46,XX karyotype. If *SEMA3A*and *AKR1C4* variants were responsible, they might exert a dominant effect that leads to masculinization – an observation that warrants further investigation.

*NR5A1* (also named *SF1*) encoding the steroidogenic factor 1 is not only important for the formation of bipotential gonads and sex determination, but also involved in the pathogenesis of hypogonadism in mice^21^. A novel missense variant of *NR5A1* (c.34C > A, p.Leu12Met) was identified in patient P31, who has a normal male karyotype with the main phenotype of small testes. However, this variant was transmitted from the unaffected mother, who is free of primary ovarian failure (OMIM#184757). The clinical significance of this missense variant is uncertain.

In conclusion, high-throughput sequencing markedly increased the diagnostic yield and showed great potential in the discovery of novel variants. 6 novel variants were identified in *WDR11, BMP4, DMRT1, SEMA3A, AKR1C4 and NR5A1*. Some of these genes are not yet generally accepted as disease-causing genes in DSD, and only few variants have been reported in human DSD patients. The identification of novel variants in DSD patients expands the pool of candidates in disease pathogenesis. For utilization in clinical diagnosis, we recommended a core set of 56 genes as initial screening panel based on ours and previous studies from other labs, which could provide sufficient diagnostic yield while save the technical effort and financial cost.

## Additional Information

**How to cite this article:** Fan, Y. *et al*. Diagnostic Application of Targeted Next-Generation Sequencing of 80 Genes Associated with Disorders of Sexual Development. *Sci. Rep.* **7**, 44536; doi: 10.1038/srep44536 (2017).

**Publisher's note:** Springer Nature remains neutral with regard to jurisdictional claims in published maps and institutional affiliations.

## Supplementary Material

Supplementary Information

## Figures and Tables

**Table 1:** List of genes, related phenotype, inheritance and coverage in the DSD panel.

No.	Gene	(O) OMIM phenotype/(L)Literature	Inheritance*	Coverage
1	AKAP2	(L) Role in the pathways regulating ovarian development^22^	—	100%
2	AKR1C1	(L)Role in metabolism of steroid hormone^23^	—	100%
3	AKR1C2	(O)46XY sex reversal 8, 614279 (3)	AR	100%
4	AKR1C4	(O)46XY sex reversal 8, modifier, 614279 (3)	AR	100%
5	AR	(O)Androgen insensitivity, 300068 (3); Hypospadias 1, X-linked, 300633 (3)	X-LR	100%
6	ATF3	(L)A hormone responsive gene in hypospadias^24^	—	100%
7	ATRX	(O)ATR-X syndrome with gonadal abnormalities, 301040 (3)	X-LD/X-LR	100%
8	BMP15	(O)Ovarian dysgenesis 2, 300510 (3); Premature ovarian failure 4, 300510 (3)	X-linked	99%
9	BMP4	(L)Mutations reported in hypospadias^18^	AD	92%
10	BMP7	(L)Downstream of androgen signaling^25^	—	100%
11	BNC2	(L)Required for ovary development and fertility in zebrafish^26^	—	100%
12	CBX2	(O)46XY sex reversal 5, 613080 (3)	AR	99%
13	CHD7	(O)CHARGE syndrome, 214800 (3); Hypogonadotropic hypogonadism 5 with or without anosmia, 612370 (3)	AD	100%
14	CUL4B	(L)Essential for spermatogenesis and male fertility in mouse model^27^	X-LR	100%
15	CYB5A	(L)Contribute to steroid hormone synthesis^28^	AR	100%
16	CYP11A1	(O)Adrenal insufficiency, congenital, with 46XY sex reversal, partial or complete, 613743 (3)	—	100%
17	DHH	(O)46XY partial gonadal dysgenesis, 607080 (3); 46XY sex reversal 7, 233420 (3)	AR/hetero	100%
18	DMRT1	(L)Role in human sex determination with male-specific expression^14^	—	93%
19	DUSP6	(O)Hypogonadotropic hypogonadism 19 with or without anosmia, 615269 (3)	AD	95%
20	ESR2	(L)Hormone receptor in gonadal development^29^	—	98%
21	FGF17	(O)Hypogonadotropic hypogonadism 20 with or without anosmia, 615270 (3)	AD	94%
22	FGF8	(O)Hypogonadotropic hypogonadism 6 with or without anosmia, 612702 (3)	—	94%
23	FGFR1	(O)Hypogonadotropic hypogonadism 2 with or without anosmia, 147950 (3)	AD	98%
24	FGFR2	(L)Downstream of SOX9 in sex determination^30^	AR/AD	100%
25	FLRT3	(O)Hypogonadotropic hypogonadism 21 with anosmia, 615271 (3)	AD	100%
26	FSHR	(O)Ovarian dysgenesis 1, 233300 (3); Ovarian hyperstimulation syndrome, 608115 (3)	AR/AD	100%
27	GATA4	(O)Testicular anomalies with or without congenital heart disease, 615542 (3);	AD	63%
28	GNRH1	(O)Hypogonadotropic hypogonadism 12 with or without anosmia, 614841 (3)	AR	100%
29	GNRHR	(O)Hypogonadotropic hypogonadism 7 without anosmia, 146110 (3)	AR	100%
30	HDAC8	(O) Wilson-Turner syndrome, 309585 (3)	X-LD	100%
31	HESX1	(O)Pituitary hormone deficiency, combined, 5, 182230 (3)	AD/AR	88%
32	HOXA4	(L)Associated with hypospadias based on GWAS study^31^	—	58%
33	HOXB6	(L)Mutations found in Chinese patients with hypospadias^18^	—	98%
34	HS6ST1	(O)Hypogonadotropic hypogonadism 15 with or without anosmia, 614880 (3)	AD	78%
35	HSD17B3	(O)Pseudohermaphroditism, male, with gynecomastia, 264300 (3)	AR	100%
36	HSD17B4	(O)Perrault syndrome 1 (with ovarian dysfunction), 233400 (3)	AR	100%
37	HSD3B2	(O)3-beta-hydroxysteroid dehydrogenase, type II, deficiency, 201810 (3)	AR	99%
38	IL17RD	(O)Hypogonadotropic hypogonadism 18 with or without anosmia, 615267 (3)	AD	94%
39	KAL1	(O)Hypogonadotropic hypogonadism 1 with or without anosmia (Kallmann syndrome 1), 308700 (3)	X-linked	92%
40	KISS1	(O)Hypogonadotropic hypogonadism 13 with or without anosmia, 614842 (3)	AR	100%
41	KISS1R	(O)Hypogonadotropic hypogonadism 8 with or without anosmia, 614837 (3);Precocious puberty, central, 1, 176400 (3)	AD/AR	66%
42	LEP	(O)Obesity, morbid, due to leptin deficiency, 614962 (3)	AR	100%
43	LHB	(O)Hypogonadotropic hypogonadism 23 with or without anosmia, 228300 (3)	AR	42%
44	LHCGR	(O)Leydig cell hypoplasia with pseudohermaphroditism, 238320 (3)	Sex-limited AD/AR	99%
45	LHFPL5	(L) Variants reported in patients of hypospadias^32^	AR	99%
46	LHX3	(O)Pituitary hormone deficiency, combined, 3, 221750 (3)	AR	92%
47	LHX9	(L)Essential gene for gonadal formation in mouse model^33^	—	96%
48	MAMLD1	(O)Hypospadias 2, X-linked, 300758 (3)	X-LR	100%
49	MAP3K1	(O)46XY sex reversal 6, 613762 (3)	AD	91%
50	MID1	(O)Opitz GBBB syndrome, type I, 300000 (3)	X-LR	100%
51	NELF	(O)Hypogonadotropic hypogonadism 9 with or without anosmia, 614838 (3)	—	75%
52	NLGN4X	(L)Deletion identified in infants with atypical sexual development^34^	X-linked	100%
53	NMT2	(L)Disruption of gene structure found in hypogonadism^35^	—	99%
54	NR0B1	(O)Adrenal hypoplasia, congenital, with hypogonadotropic hypogonadism, 300200 (3); 46XY sex reversal 2, dosage-sensitive, 300018 (3)	X-LR	97%
55	NR5A1	(O)46XY sex reversal 3, 612965 (3); Premature ovarian failure 7, 612964 (3); Adrenocortical insufficiency (3); Spermatogenic failure 8, 613957 (3)	AR/AD	99%
56	PAX2	(L)Involved in initial formation of genital tracts^36^	AD/AR	100%
57	POLR3A	(O)Leukodystrophy (with hypogonadotropic hypogonadism), 607694 (3)	AR	100%
58	POR	(O)Antley-Bixler syndrome with genital anomalies and disordered steroidogenesis, 201750 (3); Disordered steroidogenesis due to cytochrome P450 oxidoreductase, 613571 (3)	AR	86%
59	PROK2	(O)Hypogonadotropic hypogonadism 4 with or without anosmia, 610628 (3)	AD	75%
60	PROKR2	(O)Hypogonadotropic hypogonadism 3 with or without anosmia, 244200 (3)	AD	99%
61	PROP1	(O)Pituitary hormone deficiency, combined, 2, 262600 (3)	AR	100%
62	PSMC3IP	(O)Ovarian dysgenesis 3, 614324 (3)	AR	100%
63	RSPO1	(O)Palmoplantar hyperkeratosis with squamous cell carcinoma of skin and sex reversal, 610644 (3)	—	100%
64	SEMA3A	(O)Hypogonadotropic hypogonadism 16 with or without anosmia, 614897 (3)	AD	100%
65	SOX10	(L)Implicated in human 22q-linked disorders of sex development^37^	AD	98%
66	SOX2	(L)Role in Hypogonadotropic Hypogonadism^38^	AD	99%
67	SOX3	(O)Panhypopituitarism, X-linked, 312000 (3)	X-linked	97%
68	SOX9	(O)Campomelic dysplasia with autosomal sex reversal, 114290 (3)	AD	89%
69	SPRY4	(O)Hypogonadotropic hypogonadism 17 with or without anosmia, 615266 (3)	AD	100%
70	SRD5A2	(O)Pseudovaginalperineoscrotal hypospadias, 264600 (3)	AR	96%
71	SRY	(O)46XY sex reversal 1, 400044 (3); 46XX sex reversal 1, 400045 (3)	Y-Linked	100%
72	STAR	(O)Lipoid adrenal hyperplasia, 201710 (3)	AR	100%
73	TAC3	(O)Hypogonadotropic hypogonadism 10 with or without anosmia, 614839 (3)	AR	100%
74	TACR3	(O)Hypogonadotropic hypogonadism 11 with or without anosmia, 614840 (3)	AR	100%
75	TDRD7	(L)Involved in male germline development in mouse model^39^	—	100%
76	TUBB3	(L)TUBB3 syndrome (with hypogonadotropic hypogonadism)^40^	AD	71%
77	WDR11	(O)Hypogonadotropic hypogonadism 14 with or without anosmia, 614858 (3)	AD	100%
78	WNT4	(O)Mullerian aplasia and hyperandrogenism, 158330 (3)	AD	95%
79	WT1	(O) Wilms tumor 1, associated with urogenital abnormality, 194070 (3)	AD/AR	87%
80	WWOX	(L)Exonic deletion associated with 46 XY,DSD^41^	AR/AD	100%

^*^Abbreviations for inheritance mode – AR: autosomal recessive; AD: autosomal dominant; X-LD: X-linked dominant; X-LR: X-linked recessive.

**Table 2:** Clinical phenotype, karyotype, prior genetic tests and findings in the current study.

ID	EG^#^	Kar^%^	Age	Phenotype	Anosmia	Prior tests^$^	Current study^$$^
P01	M	46,XY	9y2m	Micropenis, small testes,adrenal insufficiency		**NR0B1(+)**	**NR0B1**
P02	F	46,XX	18y	No breast growth/menstrual cycle, vague left ovary(ultrasound)	−	NR0B1(−)	−
P03	M	46,XY	9y1m	Micropenis, small testes	−	KAL1(−)	**PROKR2**
P04	M	46,XY	9y3m	Cryptorchidism		NR0B1(−)	−
P05	M	46,XY	13y	Micropenis	+	NR0B1(−)	−
P06	M	46,XY	11y6m	Small testes	−	KAL1(−)	WDR11
P07	M	46,XX	4y3m	Hypospadias, inguinal testes		NR0B1(−)	BMP4
P08	M	46,XX	1y7m	Ambiguous genitalia, inguinal testes		NR0B1(−)	−
P09	M	46,XY	1y2m	Micropenis, small testes		KAL1(−), CYP21A(−), FGFR1(−)	**SRD5A2**
P10	M	46,XY	12y4m	Micropenis, normal testes	−	KAL1(−)	−
P11	M	46,XY	4y2m	Micropenis, ID, ADHD	+	KAL1(−), FGFR1(−)	−
P12	M	46,XY	2y9m	Micropenis		HESX(−), KAL1(−)	−
P13	M	46,XY	3m	Micropenis, cryptorchidism		NR0B1(−)	**GNRHR**
P14	M	46,XY	14y6m	Micropenis	−	KAL1(−)	−
P15	M	46,XY	9y11m	No palpable testes		KAL1(−)	−
P16	M	46,XY	8y5m	Micropenis		NR0B1(−), KAL1(−), FGFR1(−)	−
P17	M	46,XY	1y9m	Cryptorchidism, micropenis, bilateral inguinal testes			−
P18	M	46,XY	8y1m	Micropenis, small testes	−	KAL1(−), FGFR1(−), FGFR3(−)	−
P19	F	46,XY	15y4m	Hypoplastic labia, uterus present		SRY detected*, FGFR3(−)	DMRT1
P20	M	46,XY	3y11m	Small testes, short stature		NR0B1(−)	−
P21	M	46,XY	18y5m	Hypospadias, gynecomastia, testes with calcification		FGFR3(−)	**AR**
P22	F	46,XY	1y4m	Ambiguous genitalia, vagina present, testis on right		SRY detected*	**SRD5A2**
P23	M	46,XX	2 m	Ambiguous genitalia, labioscrotal hypertrophy, micropenis, inguinal testes		SRY not detected*	SEMA3A,AKR1C4
P24	M	46,XY	8 m	Hypospadias		SRY detected*	−
P25	M	46,XY	15y	Small testes, bifid scrotum, obesity		KAL1(−)	−
P26	M	46,XX	6 m	Ambiguous genitalia, labioscrotal hypertrophy, palpable testes			−
P27	M	46,XY	15y7m	Small testes	+	**KAL1(+)**	**KAL1**
P28	M	46,XY	3y	Hypospadias, palpable testes, micropenis		NR0B1(−), CYP21A(−)	**SRD5A2**
P29	M	46,XY	7 m	Hypospadias, palpable testes, micropenis, congenital heart defects		NR0B1(−)	−
P30	M	46,XY	NA	Micropenis, adrenal insufficiency		CYP11B1(−)	−
P31	M	46,XY	9y8m	Small testes (bilateral inequality with calcification)		NR0B1(−)	NR5A1
P32	M	46,XY	2 m	Micropenis, inguinal testes		NR0B1(−), **KAL1(+)**	**KAL1**

^#^EG-external genitalia. ^%^Kar-karyotype. Anos- anosmia. ^*^Detection of SRY fragments based on PCR amplification and electrophoresis. ^$^The results of prior single gene tests: (+) means pathogenic/likely pathogenic variants identified in the gene; (−) negative finding. ^$$^Possible causal variants identified in the current next-generation sequencing. Gene names in bold mean a diagnosis was made based on variants identified in the gene.

**Table 3:** Details of genetic variants detected in DSD patients.

I. Genetic variants in patients with a likely molecular diagnosis											
Gene	Transcript	ID	cDNA	Protein	Chr	Position	Zygosity	Origin	Max MAF*	Effect	Variant Call^#^
SRD5A2	NM_000348.3	P09	c.680G > A	p.Arg227Gln	chr2	31754395	het	De Novo	1 kG, 0.14%	Missense | reported	P (PS2, PS3, PM2)
			c.607G > A	p.Gly203Ser	chr2	31754468	het	Maternal	ExAC, 0.01%	Missense | reported	LP (PM2, PM3, PP1, PP3)
		P22	c.16C > T	p.Gln6*	chr2	31805954	hom	NA	1 kG, 0.04%	Stop_gain | reported	P (PVS1, PM2, PP1, PP3)
		P28	c.680G > A	p.Arg227Gln	chr2	31754395	het	NA	1 kG, 0.14%	Missense | reported	P (PS2, PS3, PM2)
			c.656delT	p.Phe219fs	chr2	31754418	het	NA	ExAC, 0.002%	Frameshift | D^$^	P (PVS1, PM2, PM3)
KAL1	NM_000216.2	P27	c.1267C > T	p.Arg423*	chrX	8522080	hem	Maternal	0	Stop_gain | reported	P (PVS1, PM2, PP3)
		P32	c.1270C > T	p.Arg424*	chrX	8522077	hem	Maternal	0	Stop_gain | reported	P (PVS1, PM2, PP3)
NR0B1	NM_000475.4	P01	c.871T > G	p.Trp291Gly	chrX	30326610	hem	Maternal	0	Missense | reported	LP (PM2, PM5, PP2, PP3)
GNRHR	NM_000406.2	P13	c.521A > G	p.Gln174Arg	chr4	68619533	het	Paternal	1 kG, 0.02%	Missense| P,D,D,D^$^	LP (PM2, PM3, PP2, PP3)
			c.415C > T	p.Arg139Cys	chr4	68619639	het	Maternal	0	Missense | reported	P (PS3, PM1, PM2, PM5)
AR	NM_000044.3	P21	c.2610T > G	p.Ile870Met	chrX	66943530	hem	NA	0	Missense | reported	LP (PS3, PM2, PM1, PP3)
PROKR2	NM_144773.2	P03	c.533G > C	p.Trp178Ser	chr20	5283308	het	NA	1 kG, 0.02%	Missense | reported	LP (PM1, PM2, PP2, PP3)
**II. Genetic variants in patients with undetermined molecular diagnosis**											
**Gene**	**Transcript**	**ID**	**cDNA**	**Protein**	**Chr**	**Position**	**Zygosity**	**Origin**	**Max MAF**	**Effect(prediction**^**$**^)	**Variant Call**^**#**^
WDR11	NM_018117.11	P06	c.817C > G	p.Leu273Val	chr10	122624662	het	NA	0	Missense | D,D,D,D	VUS (PM2, PP2, PP3)
BMP4	NM_001202.3	P07	c.806G > A	p.Arg269Gln	chr14	54417171	het	Paternal	1 kG, 0.04%	Missense | D,D,D,D	VUS (PM2, PP2, PP3, BS4)
DMRT1	NM_021951.2	P19	c.251A > G	p.Tyr84Cys	chr9	842089	het	Maternal	0	Missense | D,D,D,D	VUS (PM2, PP3, BS4)
SEMA3A	NM_006080.2	P23	c.487G > C	p.Glu163Gln	chr7	83689841	het	NA	0	Missense | P,P,D,D	VUS (PM2, PP3)
AKR1C4	NM_001818.3		c.773G > A	p.Arg258His	chr10	5255049	het	NA	ESP, 1.2%	Missense | P,D,D,D	VUS (PP3)
NR5A1	NM_004959.4	P31	c.34C > A	p.Leu12Met	chr9	127265641	het	Maternal	0	Missense | B,T,U,D	VUS (PM2, BS4)

^*^Max MAF in 1000 Genome, ExAC or ESP database; ^#^Variant call based on ACMG guideline with criteria listed in parentheses: P-pathogenic, LP-likely pathogenic, VUS-variant of uncertain significance. Evidence that meets ACMG criteria: Pathogenic- PVS (very strong), PS (strong), PM(moderate), PP (supporting); Benign:BS (strong). ^$^Prediction of variants effect based on in-silico tools - for frameshift variants, MutationTaster result is provided, D:disease-causing; for missense variants, results are in the order of PolyPhen, SIFT, LRT, MutationTaster. B:benign; T:tolerated; D:damaging/deleterious; P:possibly damaging; U:unknown.
