# Supplementary material for: Diagnostic Application of Targeted Next-Generation Sequencing of 80 Genes Associated with Disorders of Sexual Development
Source: Sci Rep. 2017 Mar 15;7:44536. doi: 10.1038/srep44536 (PMC5353765; doi:10.1038/srep44536)
Supplement: Supplementary Information [file srep44536-s1.pdf]

Supplementary Information

Diagnostic Application of Targeted Next-Generation Sequencing of 80 Genes Associated with Disorders of Sexual Development

Yanjie Fan[1], Xia Zhang[1], Lili Wang[1], Ruifang Wang[1], Zhuo Huang[1], Yu Sun[1], Ruen Yao[2], Xiaodong Huang[2], Jun Ye[1], Lianshu Han[1], Wenjuan Qiu[1], Huiwen Zhang[1], Lili Liang[1], Xuefan Gu[1,*], Yongguo Yu[1,*]

[1]Department of Pediatric Endocrinology/Genetics, Xinhua Hospital, Shanghai Jiao Tong University School of Medicine, Shanghai Institute for Pediatric Research, Shanghai, 200092,China.

[2]Department of Endocrinology, Shanghai Children's Medical Center, Shanghai Jiao Tong University School of Medicine, Shanghai, 200127, China.

Supplementary Table 1. Key QC metrics of next-generation sequencing

ID	TOTAL_READS	PCT_PF_READS_ALIGNED	MEAN_COVERAGE	>1X coverage	>10X coverage
P01	216731	0.994	168.750	0.982	0.968
P02	365649	0.991	272.555	0.985	0.970
P03	186557	0.977	131.517	0.982	0.963
P04	217867	0.973	146.824	0.977	0.946
P05	214946	0.994	178.877	0.985	0.964
P06	275664	0.992	179.121	0.977	0.894
P07	214215	0.987	158.431	0.979	0.961
P08	233177	0.985	169.185	0.977	0.959
P09	258010	0.987	197.076	0.980	0.960
P10	209280	0.956	145.465	0.982	0.961
P11	193041	0.960	134.071	0.980	0.948
P12	245334	0.967	178.467	0.979	0.962
P13	220286	0.976	169.258	0.979	0.955
P14	210836	0.980	162.337	0.983	0.968
P15	216663	0.976	157.427	0.982	0.960
P16	233807	0.993	182.423	0.976	0.930
P17	338526	0.991	256.028	0.976	0.951
P18	257480	0.997	210.989	0.960	0.912
P19	381739	1.000	318.812	0.968	0.930
P20	154158	0.997	127.350	0.970	0.820
P21	171815	0.987	129.775	0.974	0.943
P22	169294	1.000	130.669	0.977	0.947
P23	260135	1.000	196.421	0.979	0.956
P24	250136	1.000	170.222	0.971	0.873
P25	321739	0.992	237.911	0.980	0.944
P26	224597	0.983	160.281	0.979	0.957
P27	257672	0.984	186.105	0.980	0.958
P28	297715	0.985	235.445	0.982	0.974
P29	199373	0.986	156.715	0.982	0.968
P30	341899	0.992	280.182	0.982	0.969
P31	282595	0.995	226.562	0.983	0.968
P32	197209	0.989	152.394	0.980	0.957
**Mean**	**244317**	**0.986**	**184.614**	**0.978**	**0.947**

Supplementary Figure S1. The overlap of the sequenced genes in our study with those in another 2 similar studies

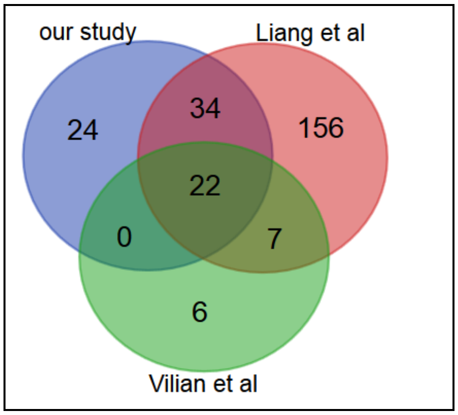

Names	total	elements
Liang et al Vilian et al our study	22	ATRX DMRT1 CYP11A1 WT1 SOX9 POR STAR LHCGR SOX3 NR0B1 AR FGFR2 MAP3K1 RSPO1 DHH CBX2 GATA4 SRD5A2 MAMLD1 NR5A1 SRY WNT4
Liang et al our study	34	BMP4 SEMA3A TACR3 HS6ST1 CUL4B CYB5A HSD3B2 KISS1 HSD17B4 POLR3A PROKR2 PAX2 GNRH1 HDAC8 FGFR1 WDR11 AKR1C4 GNRHR FGF8 LEP PSMC3IP SOX10 KAL1 LHB TAC3 HSD17B3 CHD7 PROK2 AKR1C2 MID1 SOX2 KISS1R FSHR BMP15
Liang et al Vilian et al	7	CYP17A1 INSL3 FOXL2 AMH ARX TSPYL1 DMRT2
our study	24	FLRT3 HESX1 LHFPL5 NMT2 NLGN4X TUBB3 PROP1 NELF BNC2 AKR1C1 IL17RD WWOX ESR2 DUSP6 HOXB6 LHX9 AKAP2 HOXA4 SPRY4 ATF3 BMP7 LHX3 FGF17 TDRD7
Liang et al	156	SMS ERCC8 FSHB DDX3Y KDM5D RAB3GAP2 EIF2B5 LEPR BSCL2 DMRT3 OPHN1 EIF2B3 AGPAT2 SEMA3E ALMS1 INPP5E WNT7A KLHL4 HFE UPK3A NBN TTC8 H6PD CYP19A1 UBR1 IGSF1 BBS4 SOS1 LMNA CD96 BRWD3 PITX2 ORC1 PEX5 PEX3 FRAS1 BBS2 MKS1 EIF1AY SLC39A4 PEX12 STK11 KIF7 DHCR7 INSR GPC6 RAF1 MTM1 GLI3 RECQL4 PMM2 PEX2 POLG BRCC3 EIF2B4 TSHR BLM RAB23 CREBBP ATR PCSK1 HPRT1 GK EVC ZBTB16 CUL7 HOXA13 FMR1 EIF2B1 POF1B BBS9 TBX3 ARID1B ALX4 WNT3 PEX6 OFD1 IRX5 WDPCP PRKAR1A FGD1 PCDH11Y KRAS DIAPH2 MBTPS2 BBS1 BRAF MECP2 FLNA PAPSS2 NSMF BBS10 CEP290 USP26 TBCE FREM2 USP9Y IRF6 BBS12 AMHR2 OCRL PHF6 TRIM32 STRA6 EIF2B2 WNT5A PEX1 FIGLA PTPN11 RET KDM5C HOXD13 PEX26 SLC29A3 MED12 MLL2 CYP11B1 CFTR GNAS TP63 NRAS MTCP1 GHR NSDHL MAP2K1 MKKS BBS7 PEX14 RIPK4 HCCS GATA1 BMPR1B CHRM3 EBP NAA10 BUB1B SF3B4 HARS2 SMARCA2 ROR2 ICK DCAF17 NOBOX NOTCH2 ARL6 DOCK8 DGKK NR3C1 RXFP2 RAB40AL EVC2 AIRE CDKN1C TMEM67 BBS5 HSD11B1
Vilian et al	6	CYP21A2 ZFPM2 AMHR 17BHSD3 3BHSD2 FGF9

References:

Dong Y, Yi Y, Hong Y, et al. Targeted next-generation sequencing identification of mutations in patients with disorders of sex development[J]. Bmc Medical Genetics, 2016, 17(1):1-9.

Arboleda V A, Lee H, Sánchez F J, et al. Targeted massively parallel sequencing provides comprehensive genetic diagnosis for patients with disorders of sex development[J]. Clinical Genetics, 2013, 83(1):35-43.)
